# Personalised medicine and the decision to withhold chemotherapy in early breast cancer with intermediate risk of recurrence – a systematic review and meta-analysis

**DOI:** 10.1007/s00228-020-02914-z

**Published:** 2020-06-05

**Authors:** Susanna M. Wallerstedt, Astrid Nilsson Ek, Roger Olofsson Bagge, Anikó Kovács, Annika Strandell, Barbro Linderholm

**Affiliations:** 1 HTA-centrum, Sahlgrenska University Hospital, Region Västra Götaland, Gothenburg, Sweden; 2 Department of Pharmacology, Sahlgrenska Academy, University of Gothenburg, Box 431, SE-405 30 Gothenburg, Sweden; 3 Department of Oncology, Sahlgrenska University Hospital, Gothenburg, Sweden; 4 Sahlgrenska Cancer Center, Department of Surgery, Sahlgrenska Academy, University of Gothenburg, Gothenburg, Sweden; 5 Department of Surgery, Sahlgrenska University Hospital, Gothenburg, Sweden; 6 Wallenberg Centre for Molecular and Translational Medicine, University of Gothenburg, Gothenburg, Sweden; 7 Department of Clinical Pathology, Sahlgrenska University Hospital, Gothenburg, Sweden; 8 Department of Obstetrics and Gynecology, Sahlgrenska Academy, University of Gothenburg, Gothenburg, Sweden; 9 Department of Oncology, Sahlgrenska Academy, University of Gothenburg, Gothenburg, Sweden; 10 Department of Oncology and Pathology, Karolinska Institutet, Stockholm, Sweden

**Keywords:** Breast cancer, Gene expression assay, Meta-analysis, Overall survival, Recurrence, Systematic review

## Abstract

**Purpose:**

To assess the evidence for decision making, at the health care and the patient levels, regarding the use of gene expression assays to inform chemotherapy decisions in breast cancer patients with intermediate clinical risk of recurrence.

**Methods:**

Systematic literature searches were performed (January 2002–April 2020) in Medline, Embase, PubMed, Cochrane Library, PsycINFO and HTA databases. Inclusion criteria: patients (P) were individuals with post-surgical breast cancer at intermediate clinical risk of recurrence; intervention (I)/comparison (C) was (i) use of, versus no use of, a gene expression assay and (ii) withholding versus providing chemotherapy; outcomes (O) were overall survival (OS), health-related quality of life (HRQL), and recurrence. Randomised controlled trials (RCTs) and non-RCTs were included. Random-effects meta-analyses were performed where possible.

**Results:**

Three inconclusive non-RCTs, respectively, compared OS and recurrence with and without a gene expression assay. No studies investigated HRQL. Regarding the comparison withholding versus providing chemotherapy based on a gene expression assay, one RCT and four non-RCTs evaluated OS. In the RCT, 93.9% (I) versus 93.8% (C) were alive at 9 years. Three RCTs and seven non-RCTs evaluated recurrence. Three RCTs could be pooled regarding distant recurrence; 4.29% versus 3.88% had such an event (risk ratio: 1.12 (95% confidence interval: 0.90 to 1.39).

**Conclusion:**

Regarding the use of gene expression assays in breast cancer, evidence on patient effects, informing patient-level chemotherapy decision making, is available. However, evidence for prioritisation at the overall health care level, i.e. use of, versus no use of, such assays, is largely lacking.

**Electronic supplementary material:**

The online version of this article (10.1007/s00228-020-02914-z) contains supplementary material, which is available to authorized users.

## Introduction

Personalised medicine is a twenty-first century focus. The concept implies that the drug/treatment choice for a specific patient is based on their biomarker profile. Oncology research has made important contributions within the field; precision cancer medicine aims at providing anti-cancer drugs to those who are likely to respond to the treatment and to avoid such drugs when the opposite can be expected. Indeed, these drugs are often associated with severe adverse reactions which need to be avoided if not clearly counterbalanced by beneficial effects. Furthermore, there may be great heterogeneity between tumours, and treatment may be effective only in a subset of patients. Drug development in oncology has therefore focused on defining genetic and molecular characteristics of the tumour to select patients likely to benefit from treatment [1–5].

Breast cancer, the most common cancer in women and the leading cause of cancer deaths worldwide [6, 7], has been a pioneer target for personalised medicine. The discovery of the oestrogen receptor (ER) and human epidermal growth factor receptor-2 (HER2) has enabled development of blocking therapies. In 2000–2001, the first gene expression profiling data were published, distinguishing subclasses with differences in biology and outcome [8, 9]. Subsequently, gene expression assays were developed to provide prognostic and predictive information to inform chemotherapy decision making. Several assays, covering various tumour genes, are commercially available providing information on the risk of recurrence [10]. To determine the use of this technology in health care, the benefits and risks have to be assessed. Ideally, such assessments should be based on evidence of effects on patients.

About 75% of breast cancers is hormone-sensitive (luminal) and HER2-negative [11]. Treatment decisions are based on the risk of recurrence, determined by tumour stage, and histopathological data and biomarker status as well as menopausal status [12]. For patients at intermediate risk of recurrence, decision making may be particularly difficult; there may be beneficial effects of adjuvant chemotherapy but the risks associated with such treatment are not negligible. Indeed, chemotherapy is associated with fatalities and severe, sometimes persistent, adverse reactions such as neuropathy [13–16]. Gene expression assays are included in guidelines to identify patients from whom chemotherapy can be withheld [17–19]. As far as we are aware, a summarised evidence base is currently lacking regarding patient effects of use of gene expression assays to inform chemotherapy decisions in the subgroup of patients where the clinical risk of recurrence does not suffice for clear-cut decisions. Indeed, previous systematic reviews within the field have had a wider scope [20–23]. Therefore, we performed this study to assess the evidence on critical patient effects, such as overall survival, recurrence and health-related quality of life (HRQL), of using molecular profiling to inform chemotherapy decisions in this clinically relevant patient group. This evidence is relevant in decision making at both the patient and the health care levels.

## Methods

**Fig. 1.**
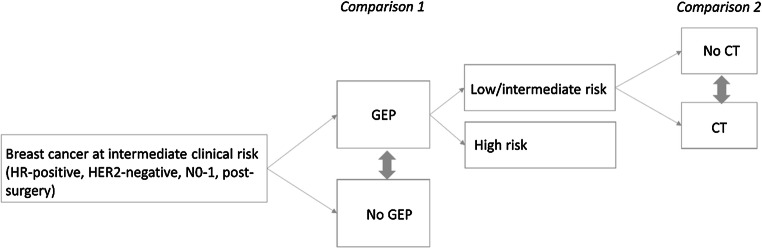
Patients and comparison groups for whom the outcomes overall survival, health-related quality of life (HRQL) and recurrence were evaluated. CT, chemotherapy; GEP, gene expression profile; HR, hormone receptor; HER2, human epidermal growth factor 2; N0-1, with no (N0) or one to three (N1) axillary lymph node metastases

We performed a systematic review according to established routines at the regional health technology assessment (HTA) centre (HTA-centrum) in Region Västra Götaland, Sweden. The aim was defined in two PICOs (Patients, Intervention, Comparison, Outcome; Fig. 1).

In the first PICO, the evidence for horizontal prioritisations was reflected i.e. the scientific basis for decision making regarding the use of gene expression assays in early breast cancer from an overall health care perspective. Patients (P) were individuals with post-surgical breast cancer at intermediate clinical risk of recurrence i.e. ER-positive, HER2-negative and with up to three axillary lymph node metastases (N0-1). The intervention (I) was a gene expression assay, including the patient management and chemotherapy decision making based on the test results. The comparison (C) was no gene expression assay, including standard patient management and chemotherapy decision making. Outcomes (O) were overall survival, HRQL and recurrence. The outcome HRQL was chosen to capture the experience of adverse effects of chemotherapy.

In the second PICO, evidence for decision making at the patient level, i.e. the scientific basis to be guided in chemotherapy decisions by a gene expression assay, was reflected. The patients were the same as those in the first PICO, namely patients in whom the clinical risk of recurrence did not suffice for clear-cut decisions, with the addition of the tumour being categorised as low/intermediate risk of recurrence based on a gene expression assay. The intervention was to withhold chemotherapy, and the comparison was to provide chemotherapy. The outcomes were the same as for the first PICO: overall survival, HRQL and recurrence.

We included both randomised controlled trials (RCTs) and non-randomised controlled trials (non-RCTs). We restricted the search to English or Scandinavian-language (Swedish, Danish and Norwegian) publications.

### Literature search and study selection

Systematic searches during August 2018, with updates in January 2019 and April 2020, covering the period from January 2002, were performed in Medline, Embase, PubMed, the Cochrane Library, PsycINFO and a number of HTA databases. Search strategies are provided in Appendix 1. Reference lists of relevant articles were scrutinised for additional references. To identify ongoing or completed but not yet published studies, we searched Clinicaltrials.gov in December 2018, with an update in April 2020.

Identified abstracts were screened by two persons and those that did not meet the PICO criteria were excluded in a consensus discussion. When there were uncertainties regarding inclusion/exclusion, the full text was retrieved. For articles excluded in consensus, after full-text reading, reasons for exclusions were recorded. The remaining studies were included in the systematic review.

### Data extraction and quality assessment

Data were extracted from the studies by one author and were subsequently checked by the other authors. Data extraction included the number of individuals in the intervention and control groups, the type of gene expression assay used and the results. When the number of events in the randomisation groups was not available in the original RCT for poolable results, the corresponding author was contacted to obtain the relevant information.

The studies were critically appraised by all authors, according to checklists from the Swedish Agency for Health Technology Assessment and Assessment of Social Services (SBU) [24]. These include assessment of three domains: directness, risk of bias and precision. The authors discussed the assessments and categorised each study as having no or minor problems (+), some problems (?) or major problems (–) in each domain. Disagreements were resolved by discussion. The certainty of evidence, i.e. the confidence in the effect estimate, was then assessed using the Grading of Recommendations Assessment, Development and Evaluation (GRADE) [25].

### Statistics

RCTs were pooled in random-effects meta-analyses using the software Review Manager (RevMan) version 5.3 (The Nordic Cochrane Centre, The Cochrane Collaboration, Copenhagen, Denmark). Heterogeneity was assessed with *I*^2^. The individual studies and the pooled estimates were presented in forest plots. Results are presented as risk ratios (RRs) and 95% confidence intervals (CIs).

## Results

**Fig. 2:**
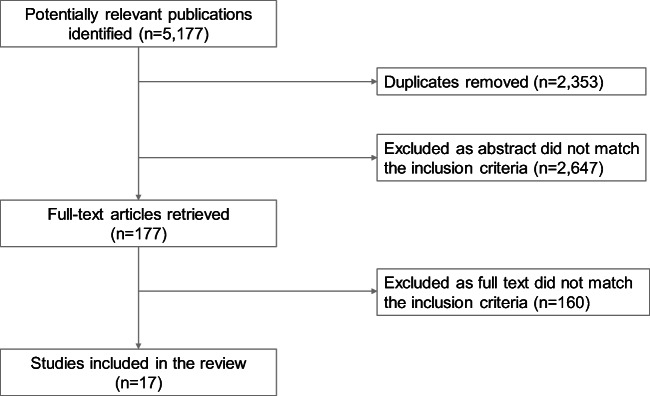
Flowchart of studies included in this systematic review

After removal of duplicates, 2,824 references were identified, 17 of which fulfilled the criteria of either of the PICOs (Fig. 2). Studies excluded after full-text reading are presented in Appendix 2.

### Study characteristics

Three RCTs and 14 non-RCTs were included in the review (Table 1). Four non-RCTs investigated patient outcome with and without a gene expression assay [26–29], while the remaining three RCTs [30–32] and ten non-RCTs [33–42] investigated withholding versus providing chemotherapy. From two RCTs, only a subset of the patients fulfilled the P criteria of this review [30, 31].

**Table 1:** Characteristics of studies fulfilling the PICO criteria. The 95% confidence interval (CI) is presented in parentheses

Author year country/region of study population	Study design	Patients (*n*)	Test	Outcome	Results (I vs C)*		Comments	Directness**	Risk of bias**	Precision**
*Comparison 1: GEP versus no GEP*										
Pomponio et al. 2020 [27] USA	Cohort	2,307 *I* = 1,149 *C* = 1.158	Oncotype DX	OS Recurrence	Average treatment effect in months at a median follow-up of 42 (I) and 44 (C) months OS: -1.63 (-12.78 to 9.51) DFS: 16.43 (4.50 to 28.38)		Focus: use of a gene expression assay to identify patients to receive chemotherapy despite favourable histopathology variables Patients in the intervention group were younger and had more advanced cancer IPW based on PS to adjust for imbalances	?	-	?
Rath et al. 2018 [26] Germany	Cohort	88 *I* = 44 *C* = 44	Oncotype DX	Recurrence	R at a mean follow-up of 19.7 months: 2/44 (4.5%) vs 0/44 (0.0%)		Matched for stage, tumour grade, menopausal and hormone receptor status Of the two cases with recurrence one patient rejected recommended chemotherapy and one withdrew from endocrine therapy because of side effects	+	–	–
Thibodeau et al. 2019 [28] Canada	Historic control	361 *I* = 201 *C* = 160	Oncotype DX	OS Recurrence	Deaths at a mean follow-up of 33.9 (I) and 87.3 (C) months 7/201 (3.5%) vs 28/160 (17.5%) Between-groups comparison: P = 0.83 R: 3/201 (1.5%) vs 11/160 (6.9%) Between-groups comparison: P = 0.35		Unmatched groups Characteristics of compared groups differed No adjustments	?	–	–
Zhang et al. 2020 [29] USA	Cohort	N0 47,040 *I* = 23,520 *C* = 23.520 N1 10,578 *I* = 5,289 *C* = 5,289	Oncotype DX	OS	OS at a median follow-up of 38 (node-) and 35 (node+) months HR_death, node-_ 0.49 (0.441 to 0.55) HR_death, node+_ 0.58 (0.48 to 2.44)		PS-matched groups, based on sociodemographic factors and tumour characteristics Characteristics of compared groups not reported Unclarity regarding the number of patients included in the analysis Survival benefit not consistent across node+ groups	?	–	+
*Comparison 2: no CT versus CT*										
Cardoso et al. 2016 [30] Europe	RCT, subset	699 *I* = 350 *C* = 349	MammaPrint	Recurrence	DRFS at 5 yrs: Low genetic risk 93.9% (90.6 to 96.1%) vs 95.5% (92.5 to 97.3%) HR_DR_ 1.25 (0.69 to 2.25)		Non-inferiority design in the main study. Subset with high clinical /low genetic risk	+	+	–
Geyer et al. 2018 [31] USA	RCT, subset	447 *I* = 169 *C* = 278	Oncotype DX	Recurrence	DR at 10 yrs: RS ≤ 25 11/169 (6.5%) vs 16/278 (5.8%) HR_DR, RS ≤ 10_ 0.84 (0.28 to 2.44) HR_DR, RS11-25_ 1.64 (0.74 to 3.85)		Analysis of a subset of an RCT 2,363 patients, 1988–1993, given treatment with or without chemotherapy, including patients with an RS score available and excluding HER2+ individuals.	?	–	?
Sparano et al. 2018 [32] USA	RCT	Randomised: 6,907 *I* = 3,458 *C* = 3,449 In analysis: 6,711 *I* = 3,399 *C* = 3,312	Oncotype DX	OS Recurrence	RS 11–25 OS at 9 yrs: 93.9% (92.9 to 94.9%) vs 93.8% (92.8 to 94.8%) HR_death_ 0.99 (0.79 to 1.22) DRFS at 9 yrs: 94.5% (93.5 to 95.5%) vs 95.0% (94.0 to 96.0%) HR_DR_ 1.10 (0.85 to 1.41) RFS at 9 yrs: 92.2% (91.0 to 93.4%) vs 92.9% (91.7 to 94.1%) HR_recurr_ 1.11 (0.90 to 1.37)		Non-inferiority design. Margin set at 32.2% higher risk of the composite outcome invasive disease recurrence, second primary cancer or death when calculating HR, accepting 87% invasive disease-free survival without chemotherapy compared with 90% with chemotherapy.	?	?	+?
Barcenas et al. 2017 [33] USA	Cohort	549 *I* = 457 *C* = 92 178 *I* = 89 *C* = 89	Oncotype DX	OS Recurrence,	Unmatched cohort RS 11–25 OS at 5 yrs: 98% (96 to 99%) vs 98% (91 to 99%) HR_death_ 0.46 (0.09 to 2.72) RFS at 5 yrs: 96% (94 to 98%) vs 95% (86 to 98%) HR_recurr_ 0.68 (0.19 to 2.44) Matched cohort RS 18–30 HR_recurr_ 1.02 (0.33 to 3.13) Matched cohort RS 18–30 HR_death_ 1.16 (0.20 to 6.67)		In unmatched analyses: patients in the control group (receiving chemotherapy) were younger and had more advanced cancer.	+	?	–
Chen et al. 2018 [34] USA	Cohort	21,991 *I* = 17,345 *C* = 4,646	Oncotype DX	OS	RS 11–25 OS at 5 yrs: 97.6% (96.9 to 98.2%) vs 97.4% (95.3 to 98.5%) HR_death_ 0.83 (0.55 to 1.25)		Unmatched groups Patients in the control group (receiving chemotherapy) were younger and had more advanced cancer.	?	–	?
Ibraheem et al. 2019 [35] USA	Cohort	73,185 (unmatched) *I* = 55,327 *C* = 17,858 27,740 (matched) *I* = 13,735 *C* = 13,735	Oncotype DX	OS	Unmatched cohort RS 11–30 HR_death, node-_ 1.18 (0.99 to 1.41) HR_death, node+_ 1.72 (1.35 to 2.22) Matched cohort, RS 11–30 HR_death, node-_ 1.33 (1.09 to 1.67) HR_death, node+_ 1.92 (1.43 to 2.56)		Unmatched groups Patients receiving chemotherapy were younger and had more advanced cancer. Characteristics of compared groups not reported in matched cohort.	?	+	+
Le Du et al. 2015 [36] USA	Cohort	341 *I* = 189 *C* = 152	Oncotype DX	Recurrence	RS 18–30 DR at a median follow-up of 3.2 yrs: 10/189 (5.3%) vs 16/152 (10.5%)		Unmatched groups Patients in the control group (receiving chemotherapy) were younger and had more advanced cancer.	+	–	–
Park et al. 2019 [37] USA	Cohort	3,540 *I* = 1,438 *C* = 2,102	Oncotype DX	OS	RS 26–30, ≤70 years old, At a mean follow-up of 32 months (in the whole cohort, also including 19,791 patients with RS 18-25) HR_death_ 1.39 (0.88 to 2.22)		Unmatched groups Patients in the control group (receiving chemotherapy) were younger and had more advanced cancer Age- and clinic-pathological and treatment factor-adjusted model	?	?	?
Sestak et al. 2019 [38] Europe	Cohort	NR	EndoPredict	Recurrence	10 year risk of DR according to EndoPredict clinical scores indicating low risk (score 1-3) Score 1: 1.0% (0.6 to 1.4) vs 1.1% (0.5 to 1.7) Score 2: 2.8% (2.1 to 3.5) vs 2.5% (1.5 to 3.5) Score 3: 7.6% (6.4 to 8.8) vs 5.7% (4.1 to 7.2)		Based on data from five clinical trials Unmatched groups Characteristics of compared groups within the genomic low risk population not reported	–	–	–
Stemmer et al. 2017 [41] Israel	Cohort	562 *I* = 473 *C* = 89	Oncotype DX	Recurrence	RS 18–25 DR at a median follow-up of 6.2 yrs: 17/473 (3.6%) vs 5/89 (5.6%); P = 0.434		Node-. Unmatched groups Patients in the control group (receiving chemotherapy) were younger and had more advanced cancer.	?	–	?
Stemmer et al. 2017 [40] Israel	Cohort	637 *I* = 508 *C* = 129	Oncotype DX	Recurrence	RS ≤ 25 DR at 5 yrs: 21/488 (4.4%) vs 2/89 (2.3%); P = 0.521		Node+. Unmatched groups Characteristics of compared groups not reported	+	–	–
Stemmer et al. 2019 [39] Israel	Cohort	853 *I* = 773 *C* = 80	Oncotype DX	Recurrence	RS 11-25 DR at a median follow-up of 9 years: 34/773 (4.4%) vs 6/80 (10%) (P = 0.703)		Unmatched groups Characteristics of compared groups not reported	?	–	–
Wen et al. 2016 [42] USA	Cohort	1,406 *I* = 1,236 *C* = 170	Oncotype DX	Recurrence	RS < 18 DR at a median follow-up of 46 months: 5/1,236 (0.4%) vs 1/170 (0.6%)		Unmatched groups Characteristics of compared groups not reported	+	–	–

*We inverted the HR provided in the publication if their analysis presented results for control versus intervention; 95% CI provided within parentheses.** + =no or minor problems; ? = some problems; – = major problems

*C* control, *CI* confidence interval, *CT* chemotherapy, *DFS* disease-free survival, *DR* distant recurrence, *DRFS* distant recurrence-free survival, *GEP* gene expression profile, *HR* hazard ratio, *I* intervention, *IDFS* invasive disease-free survival (freedom from invasive disease recurrence, second primary cancer and death), *IPW* inverse probability weighting, *NR* not reported, *OS* overall survival, *PS* propensity score, *R* recurrence, *RCT* randomised controlled trial, *RFS* recurrence-free survival, *RS* recurrence score, *UK* United Kingdom, *yrs* years

### Gene expression assay versus no gene expression assay

No RCT and four non-RCTs [26–29] reported results regarding overall survival and/or recurrence in patients with intermediate clinical risk of recurrence where a gene expression assay had been, versus had not been, performed. No studies evaluated effects on HRQL.

#### Overall survival

Three studies reported results regarding overall survival, using a cohort design [27, 29] or a historic control [28], and including a total of 60,286 patients. Results favouring the use of a gene expression assay were reported in one study [29], whereas the other two studies reported no difference [27, 28]. The certainty of evidence was downgraded one step as the characteristics of the compared groups either differed [27, 28] or were not reported [29]. In summary, it is uncertain whether use of, versus no use of, a gene expression assay affects overall survival in breast cancer patients with intermediate clinical risk of recurrence (very low certainty of evidence, GRADE ⊕◯◯◯).

#### Recurrence

Three studies reported results regarding recurrence, using a cohort design [26, 27] or a historic control [28], and including a total of 2,756 patients. One study reported prolonged time to recurrence when a gene expression assay had been used [27], whereas the other two studies reported no difference [26, 28]. The certainty of evidence was downgraded one step as the characteristics of the compared groups differed, and there was some uncertainty about the directness and the precision. In summary, it is uncertain whether use of, versus no use of, a gene expression assay affects recurrence in breast cancer patients with intermediate clinical risk of recurrence (very low certainty of evidence, GRADE ⊕◯◯◯).

### Withholding versus providing chemotherapy

Three RCTs and ten cohort studies investigated withholding versus providing chemotherapy regarding the overall survival and/or recurrence in patients with intermediate clinical risk of recurrence and low/intermediate genetic risk of recurrence. No studies evaluated effects on HRQL.

#### Overall survival

Overall survival was reported in one RCT [32] and four cohort studies [33–35, 37]. In the RCT, including 6,711 patients, similar overall survival rates were found at 9 years: 93.9% and 93.8% in the intervention and comparison groups, respectively [32]. Loss to follow-up differed between the comparison groups and was large in relation to the number of events. Three cohort studies reported non-significant results [33, 34, 37], whereas the remaining study reported significantly better outcome for patients who had been administered chemotherapy [35]. In summary, withholding adjuvant chemotherapy to breast cancer patients with intermediate clinical risk of recurrence and low/intermediate risk according to a gene expression assay, compared with providing chemotherapy, probably results in little or no difference in medium-term survival (moderate certainty of evidence, GRADE ⊕⊕⊕◯).

#### Recurrence

**Fig. 3:**
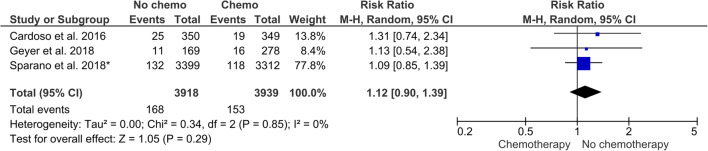
Meta-analysis of randomised controlled trials (RCTs) comparing withholding versus providing adjuvant chemotherapy regarding distant recurrence. Single asterisk indicates number of events obtained from the corresponding author. CI, confidence interval; M-H, Mantel-Haenszel

Three RCTs and seven cohort studies reported data on recurrence. The RCT results regarding distant recurrence could be pooled in a meta-analysis; 4.29% and 3.88% of the patients had such an event when chemotherapy was not offered and offered, respectively, based on the results of a gene expression assay. The absolute risk difference was 0.41 percentage points (95% CI: − 0.54 to 1.36). The RR for a distant recurrence was 1.12 (95% CI: 0.90 to 1.39; *I*^2^ = 0) (Fig. 3).

Five out of seven cohort studies reported the number of distant recurrences in the comparison groups [36, 39–42]. In four of these, numerically more patients who had been provided chemotherapy had such an event [36, 39, 41, 42]. The remaining two studies reported either a non-significant hazard ratio with a wide CI [33] or overlapping CIs between the 10-year risk of recurrence of the comparison groups [38]. In three out of the seven cohort studies, patients in the control group were younger and had more advanced cancer [33, 36, 41]. The remaining four studies did not present characteristics of the compared groups [38–40, 42].

In summary, withholding adjuvant chemotherapy in breast cancer patients with intermediate clinical risk of recurrence and low/intermediate risk based on a gene expression assay, compared with providing chemotherapy, can probably not exclude a small absolute increased risk of recurrence (moderate certainty of evidence, GRADE ⊕⊕⊕◯).

### Ongoing studies

Out of 155 ongoing/completed unpublished studies identified in Clinical Trials, one study completed in 2009 fulfilled the PICO criteria comparing use of a gene expression assay versus no gene expression assay and using a retrospective cohort design (NCT00904566). Two studies, estimated to be completed in 2026 and 2031, respectively, may contribute information regarding the PICO comparing withholding versus providing chemotherapy using a prospective cohort design (NCT03904173, NCT03503799).

## Discussion

Our review shows that evidence is largely lacking regarding patient effects of use of, versus no use of, a gene expression assay to inform chemotherapy decisions. Such evidence is useful for prioritisation at the overall health care level. However, there is probably little or no difference in medium-term overall survival when chemotherapy is withheld based on a gene expression assay. Nevertheless, it cannot be ruled out that withholding chemotherapy based on such a test implies an increased risk of recurrence, although the absolute risk is low and the absolute risk difference is small. Given this evidence base, some important knowledge gaps still exist with respect to the use of gene expression assays in breast cancer i.e. personalised medicine. These gaps need to be addressed to inform assessments of the benefit-risk balance.

For diagnostic tools sensitivity and specificity may be the primary issue. For molecular profiling in breast cancer, this has been the main research question [22]. However, as precision cancer medicine is emerging, and the upcoming diagnostic tests imply non-negligible costs, one may argue that scientific evaluations regarding patient effects should be designed and performed to provide a scientific basis for prioritisation.

For technologies to inform chemotherapy decisions, it may be particularly important to evaluate potential effects on HRQL. On the one hand, withholding chemotherapy may increase HRQL because of avoided adverse reactions. On the other, increased fear of recurrence may decrease HRQL [43]. Indeed, to introduce a technology which in itself is costly when available evidence is restricted to negative effects may be problematic. It may be argued that the introduction of gene expression assays will reduce the provision of chemotherapy, thereby reducing the costs to justify a potential worse patient outcome. However, chemotherapy decision making studies in the relevant patient group have reported both increased [44–47] and decreased [48–51] administration of chemotherapy when gene expression assay results are provided, and none of these studies had a randomised design. Also, from an ethical perspective, withholding an established treatment may be more problematic than introducing a new one. Therefore, this may call for a more solid evidence base. Conversely, new cancer drugs are sometimes approved based on limited evidence regarding patient-relevant effects [52], and not all meet the threshold for a clinically meaningful effect [53].

Diagnostic tests are used in therapy decision making at the patient level. Most breast cancer patients want to have an active or shared role in decision making regarding chemotherapy [54], as also illustrated in the largest RCT included in this review where the recruitment of patients had to be increased by 73% as 12% of the women chose not to adhere to the assigned treatment [32]. Given the results in the present review, it may be surprising that the guidelines update in 2017 [55], but not in 2019 [17], emphasised that node-positive patients should be informed of the potential benefits from chemotherapy. Indeed, an internationally used “objective” test result may have a large impact on chemotherapy decision making, which is illustrated by the fact that several studies have been performed in patients with intermediate clinical risk of recurrence, in which the chemotherapy decision was based solely on the results of the gene expression assay [26, 45, 56]. Conversely, our results suggest that many oncologists and patients take clinical parameters into account, also when the gene expression assay shows a low/intermediate risk of recurrence. In fact, several cohort studies in this review report that patients given chemotherapy, despite a low/intermediate risk of recurrence according to the gene expression assay, were younger and had more advanced disease [33, 36, 41]. As chemotherapy per se is not likely to increase the risk of recurrence, this finding may also explain that a greater number of distant recurrences occurred in those receiving chemotherapy in cohort studies [36, 41, 42].

In patients with intermediate clinical risk of recurrence and low/intermediate risk of recurrence according to a gene expression assay, the difference between withholding and providing chemotherapy was not statistically significant. However, the confidence interval was quite wide, including an up to 39% increased relative risk of a distant recurrence. To facilitate the process of informing the patient and to contribute to informed decision making, the absolute risk estimate provided in this review may be useful. The mean absolute risk increase of 0.41 percentage points regarding distant recurrence would yield in a number needed to treat (NNT) of 244. Furthermore, the upper confidence limit, of particular interest when investigating non-inferiority, was a 1.36 percentage point increase, yielding a minimum NNT of 74. Consequently, at the minimum, 74 breast cancer patients would have to endure adverse reactions from chemotherapy to avoid one distant recurrence.

As patients live many years after a breast cancer diagnosis, as illustrated by the fact that 94% was still alive after 9 years in the main RCT [32], it would take a long time to achieve mature data on long-term survival when a gene expression assay is used to guide treatment decisions. Indeed, the risk of distant recurrence and death from oestrogen dependent breast cancer persists over at least 20 years, also in low-risk patients [57]. Analyses of register data may contribute valuable information in the meantime, in particular as our evidence synthesis shows that an increased risk, although small in absolute numbers, of distant recurrence cannot be excluded if chemotherapy is withheld based on genetic testing. However, as current drug effectiveness and safety studies often have major methodological problems [58, 59], scientific rigour in the design and reporting will be crucial. For example, efforts have to be made to balance the comparison groups with respect to the severity of disease. Indeed, where data on characteristics of the comparison groups were available in the cohort studies in this review, patients administered chemotherapy had more advanced cancer. It is noteworthy that the one study with minor study limitations evaluating overall survival in matched comparisons reported better outcomes for those treated with chemotherapy [35].

Multivariable analysis may provide information on the association between various factors and patient outcome. Unfortunately, none of the studies in this review which performed such analyses included provision of chemotherapy in the analysis [34–38, 40, 41]. Although pharmacoepidemiological studies should ideally be specifically designed to evaluate drug effects [58, 59], inclusion of the provision of chemotherapy would be of interest. Importantly, causality cannot be claimed in such analyses; a cross-sectional design would be applied although seemingly mimicking a cohort design [58].

### Strengths and limitations

The main strength of this systematic review and meta-analysis is that it gives an overview of the compiled current evidence on patient effects using gene expression assays in the subgroup of breast cancer patients where the clinical risk of recurrence does not suffice for clear-cut decisions. In addition, the findings are discussed in a wider context, which is of relevance for decision making at both the health care and the patient levels and for future research within personalised medicine. Indeed, precision cancer medicine is a rapidly growing field.

Limitations include that few studies fulfilled our PICO criteria, in particular regarding the comparison of patient effects of use versus no use of a gene expression assay. Furthermore, the CI for the RR in the meta-analysis, comparing withholding versus providing chemotherapy, was fairly wide, ranging from 10% decreased to 39% increased risk of distant recurrence. Translated to absolute numbers, the risk variation was small, from 0.5% decreased risk to 1.4% increased risk. Nevertheless, the as-treated analysis in the largest RCT showed superiority for the primary composite outcome (invasive-disease recurrence, second primary cancer or death) for the randomisation group allocated to chemotherapy, according to the predetermined statistical non-inferiority definitions [32], supporting the conclusion that an increase in recurrence cannot be excluded in those not allocated to chemotherapy.

## Conclusion

In summary, this systematic review and meta-analysis illustrates that the evidence base for decision making at the overall health care level regarding the use of a gene expression assay to guide chemotherapy decisions in breast cancer with intermediate risk of recurrence is still limited. For decision making at the patient level, on the other hand, evidence is more solid; withholding chemotherapy based on the results of such a genetic tumour test probably yields similar chances of medium-term survival, but an increased risk of recurrence, though small in absolute numbers, cannot be excluded. As breast cancer research may be considered fairly advanced within the field of personalised medicine, our results may encourage an increased focus in precision cancer medicine to contribute evidence essential for horizontal prioritisation i.e. a scientific basis for assessments of the overall benefit-risk balance.

## Electronic supplementary material

ESM 1 Search strategies (DOCX 29 kb)

ESM 2 Studies excluded after full-text reading by the authors, as well as the reason for excluding them (DOCX 48 kb)

## Data Availability

All data analysed during the current study are provided in the article and in the supplement.
