# Supplementary material for: Personalised medicine and the decision to withhold chemotherapy in early breast cancer with intermediate risk of recurrence – a systematic review and meta-analysis
Source: Eur J Clin Pharmacol. 2020 Jun 5;76(9):1199–211. doi: 10.1007/s00228-020-02914-z (PMC7419442; doi:10.1007/s00228-020-02914-z)
Supplement: Supplementary file 1 — Search strategies (DOCX 29 kb) [file 228_2020_2914_MOESM1_ESM.docx]

**Appendix 1** Search strategies

**Database: Ovid MEDLINE(R) ALL**1946 to April 15, 2020

**Date:** First search on Aug 16 2018, updates on Jan 30 2019 and April 17 2020
**No of results: 1,952**

| **#** | **Searches** | **Results** |
| --- | --- | --- |
| 1 | ((breast or breasts or mammary or mamma or ductal) adj3 (neoplasm* or tumor* or tumour* or carcinoma* or cancer* or malignan* or sarcoma* or adenocarcinoma* or adeno carcinoma*)).ab,ti. | 354,947 |
| 2 | exp Breast Neoplasms/ | 288,695 |
| 3 | 1 or 2 | 414,240 |
| 4 | (multigene or multi-gene or nanostring or pam50 or prosigna or endopredict or oncotype* or mammaprint or mamma-print or 70-gene or seventy gene or 21-Gene or twenty-one gene or 12-gene or 50-gene or earlyR or recurrence score or agendia or genomic health or myriad genetics or breast cancer index or biotheranostics).ab,ti. | 13,334 |
| 5 | 3 and 4 | 2,057 |
| 6 | (animals not (animals and humans)).sh. | 4,657,092 |
| 7 | (animal or animals or rat or rats or mouse or mice or dog or dogs or cat or cats or hamster or hamsters or rabbit or rabbits or swine or sheep or cattle).ti. | 1,853,301 |
| 8 | 6 or 7 | 4,984,540 |
| 9 | 5 not 8 | 2,028 |
| **10** | **limit 9 to (yr="2002 -Current" and (danish or english or norwegian or swedish))** | **1,952** |

**Database: Embase**1974 to 2020 April 15

**Date:** First search on Aug 16 2018, updates on Jan 30 2019 and April 17 2020

**No of results: 2,264**

| **#** | **Searches** | **Results** |
| --- | --- | --- |
| 1 | ((breast or breasts or mammary or mamma or ductal) adj3 (neoplasm* or tumor* or tumour* or carcinoma* or cancer* or malignan* or sarcoma* or adenocarcinoma* or adeno carcinoma*)).ab,ti. | 498,685 |
| 2 | breast tumor/ or exp breast cancer/ | 522,838 |
| 3 | 1 or 2 | 615,232 |
| 4 | (multigene or multi-gene or nanostring or pam50 or prosigna or endopredict or oncotype* or mammaprint or mamma-print or 70-gene or seventy gene or 21-Gene or twenty-one gene or 12-gene or 50-gene or earlyR or recurrence score or agendia or genomic health or myriad genetics or breast cancer index or biotheranostics).dm,dv,mv,my. | 637 |
| 5 | (multigene or multi-gene or nanostring or pam50 or prosigna or endopredict or oncotype* or mammaprint or mamma-print or 70-gene or seventy gene or 21-Gene or twenty-one gene or 12-gene or 50-gene or earlyR or recurrence score or agendia or genomic health or myriad genetics or breast cancer index or biotheranostics).ab,kw,ti. | 21,044 |
| 6 | 4 or 5 | 21,288 |
| 7 | 3 and 6 | 5,382 |
| 8 | (animal not (animal and human)).sh. | 1,063,298 |
| 9 | (animal or animals or rat or rats or mouse or mice or dog or dogs or cat or cats or hamster or hamsters or rabbit or rabbits or swine or sheep or cattle).ti. | 1,936,845 |
| 10 | 8 or 9 | 2,746,279 |
| 11 | 7 not 10 | 5,359 |
| 12 | limit 11 to ((danish or english or norwegian or swedish) and yr="2002 -Current") | 5,219 |
| 13 | limit 12 to (embase or medline) | 2,372 |
| **14** | **limit 13 to (article or article in press or chapter or conference paper or note or "review" or short survey)** | **2,264** |

**Database:** The Cochrane Library

**Date:** First search on Aug 16 2018, updates on Jan 30 2019 and April 17 2020

**No of results:** 354

| **ID** | **Search** | **Hits** |
| --- | --- | --- |
| #1 | ((breast or breasts or mammary or mamma or ductal) NEAR/3 (neoplasm* or tumor* or tumour* or carcinoma* or cancer* or malignan* or sarcoma* or adenocarcinoma* or adeno carcinoma*)):ti,ab,kw (Word variations have been searched) | 35,962 |
| #2 | MeSH descriptor: [Breast Neoplasms] explode all trees | 12,651 |
| #3 | #1 OR #2 | 35,962 |
| #4 | (multigene OR "multi gene" OR nanostring OR pam50 OR prosigna OR endopredict OR oncotype* OR mammaprint OR "mamma print" OR "70 gene" OR "seventy gene" OR "21 Gene" OR "twenty one gene" OR "12 gene" OR "50 gene" OR earlyR “recurrence score” OR agendia OR "genomic health" OR "myriad genetics" OR "breast cancer index" OR biotheranostics):ti,ab,kw (Word variations have been searched) | 761 |
| #5 | #3 AND #4 | 406 |
| #6 | (clinicaltrials or trialsearch):so | 324,914 |
| **#7** | **#5 NOT #6** | **354** |

**Database:** APA PsycInfo, EBSCOhost Research Databases

**Date:** First search on Aug 16 2018, updates on Jan 30 2019 and April 17 2020

**No of results:** 27

| **#** | **Undran** | **Resultat** |
| --- | --- | --- |
| **S5** | **S1 AND S4** | **27** |
| S4 | S2 OR S3 | 14,335 |
| S3 | DE "Breast Neoplasms" | 11,224 |
| S2 | TI ( (breast or breasts or mammary or mamma or ductal) N3 (neoplasm* or tumor* or tumour* or carcinoma* or cancer* or malignan* or sarcoma* or adenocarcinoma* or adeno carcinoma*) ) OR AB ( (breast or breasts or mammary or mamma or ductal) N3 (neoplasm* or tumor* or tumour* or carcinoma* or cancer* or malignan* or sarcoma* or adenocarcinoma* or adeno carcinoma*) ) | 13,310 |
| S1 | TI ( multigene OR multi-gene OR nanostring OR pam50 OR prosigna OR endopredict OR oncotype* OR mammaprint OR mamma-print OR 70-gene OR "seventy gene" OR 21-Gene OR "twenty-one gene" OR 12-gene OR 50-gene OR earlyR OR “recurrence score” OR agendia OR "genomic health" OR "myriad genetics" OR "breast cancer index" OR biotheranostics ) OR AB ( multigene OR multi-gene OR nanostring OR pam50 OR prosigna OR endopredict OR oncotype* OR mammaprint OR mamma-print OR 70-gene OR "seventy gene" OR 21-Gene OR "twenty-one gene" OR 12-gene OR 50-gene OR earlyR OR “recurrence score” OR agendia OR "genomic health" OR "myriad genetics" OR "breast cancer index" OR biotheranostics ) | 160 |

**Database:** PubMed

**Date:** First search on Aug 16 2018, updates on Jan 30 2019 and April 17 2020

**No of results: 505**

| **Search** | **Query** | **Items found** |
| --- | --- | --- |
| [**#8**](https://www.ncbi.nlm.nih.gov/pubmed) | **Search #7 AND #1** | [**505**](https://www.ncbi.nlm.nih.gov/pubmed/?cmd=HistorySearch&querykey=8) |
| [#7](https://www.ncbi.nlm.nih.gov/pubmed) | Search #5 AND #6 | [2283](https://www.ncbi.nlm.nih.gov/pubmed/?cmd=HistorySearch&querykey=7) |
| [#6](https://www.ncbi.nlm.nih.gov/pubmed) | Search (multigene[tiab] OR multi-gene[tiab] OR nanostring[tiab] OR pam50[tiab] OR prosigna[tiab] OR endopredict[tiab] OR oncotype*[tiab] OR mammaprint[tiab] OR mamma-print[tiab] OR 70-gene[tiab] OR seventy gene[tiab] OR 21-Gene[tiab] OR twenty-one gene[tiab] OR 12-gene[tiab] OR 50-gene[tiab] OR earlyR[tiab] Or recurrence score[tiab] OR agendia[tiab] OR genomic health[tiab] OR myriad genetics[tiab] OR breast cancer index[tiab] OR biotheranostics[tiab]) | [17,175](https://www.ncbi.nlm.nih.gov/pubmed/?cmd=HistorySearch&querykey=6) |
| [#5](https://www.ncbi.nlm.nih.gov/pubmed) | Search #3 OR #4 | [437,197](https://www.ncbi.nlm.nih.gov/pubmed/?cmd=HistorySearch&querykey=5) |
| [#4](https://www.ncbi.nlm.nih.gov/pubmed) | Search "Breast Neoplasms"[Mesh] | [288,841](https://www.ncbi.nlm.nih.gov/pubmed/?cmd=HistorySearch&querykey=4) |
| [#3](https://www.ncbi.nlm.nih.gov/pubmed) | Search (breast[tiab] OR breasts[tiab] OR mammary[tiab] OR mamma[tiab] OR ductal[tiab]) AND (neoplasm*[tiab] OR tumor[tiab] OR tumors[tiab] OR tumour*[tiab] OR carcinoma*[tiab] OR cancer*[tiab] OR malignan*[tiab] OR sarcoma*[tiab] OR adenocarcinoma*[tiab] OR adeno carcinoma*[tiab]) | [389,799](https://www.ncbi.nlm.nih.gov/pubmed/?cmd=HistorySearch&querykey=3) |
| [#1](https://www.ncbi.nlm.nih.gov/pubmed) | Search pubmednotmedline[sb] OR inprocess[sb] OR publisher[sb] | [4,162,392](https://www.ncbi.nlm.nih.gov/pubmed/?cmd=HistorySearch&querykey=1) |

**Database:** CRD

**Date:** Aug 16 2018, no updates as this database stopped adding new records

**No of results:** 63

| **Line** | **Search** | **Hits** |
| --- | --- | --- |
| 1 | (((breast or breasts or mammary or mamma or ductal) AND (neoplasm* or tumor* or tumour* or carcinoma* or cancer* or malignan* or sarcoma* or adenocarcinoma* or adeno carcinoma*))) | 2,511 |
| 2 | MeSH DESCRIPTOR breast neoplasms EXPLODE ALL TREES | 1,798 |
| 3 | #1 OR #2 | 2,511 |
| 4 | (multigene OR multi-gene OR nanostring OR pam50 OR prosigna OR endopredict OR oncotype* OR mammaprint OR mamma-print OR 70-gene OR seventy gene OR 21-Gene OR twenty-one gene OR 12-gene OR 50-gene OR earlyR OR agendia OR genomic health OR myriad genetics OR breast cancer index OR biotheranostics) | 74 |
| **5** | **#3 AND #4** | **63** |

The web-sites of **SBU** and **Folkehelseinstituttet** were visited

2018-08-16

Two references relevant to the question at issue were found at SBU, nothing relevant was found at Folkehelseinstituttet.

**Reference lists**

A comprehensive review of reference lists brought 12 new records
