# Supplementary material for: Personalised medicine and the decision to withhold chemotherapy in early breast cancer with intermediate risk of recurrence – a systematic review and meta-analysis
Source: Eur J Clin Pharmacol. 2020 Jun 5;76(9):1199–211. doi: 10.1007/s00228-020-02914-z (PMC7419442; doi:10.1007/s00228-020-02914-z)
Supplement: Supplementary file 2 — Studies excluded after full-text reading by the authors, as well as the reason for excluding them (DOCX 48 kb) [file 228_2020_2914_MOESM2_ESM.docx]

**Appendix 2** Studies excluded after full-text reading, as well as the reason for excluding them

C = comparison, ER = oestrogen receptor, HER = human epidermal growth factor receptor, I = intervention, O = outcome, P = patients

| **Author, year** | **Reason for exclusion** |
| --- | --- |
| Aalders 2017 | Wrong I/C: multifocal versus focal breast cancer |
| Adamo 2019 | O missing |
| Ademuyiwa 2011 | Wrong P: includes both clinical low and high risk breast cancer |
| Albanell 2012 | Wrong P: includes clinical low risk breast cancer |
| Albanell 2016 | No C |
| Altman 2018 | Wrong P: includes HER2-positive |
| Amro 2019 | O not presented according to I/C |
| Arima 2019 | Wrong O: correlation |
| Asad 2008 | No C |
| Baitchu 2017 | Wrong publication type: conference abstract |
| Bargallo 2015 | Wrong O: treatment decision making |
| Bartlett 2019 | Wrong I: extended endocrine therapy |
| Bear 2017 | Wrong P: not suitable for surgery |
| Bhargava 2020 | Wrong P: disconcordance expected/actual Oncotype DX RS score |
| Bhutiani 2019 | Wrong O: treatment decision making |
| Biroschak 2013 | Wrong O: treatment decision making |
| Blok 2018 | Systematic review, poorly defined population |
| Bonneterre 2016 | Wrong O: treatment decision making |
| Bradbury 2020 | Wrong P: information on ER and HER2 missing |
| Breaux 2018 | Wrong O: downstaging |
| Brownlie 2015 | Wrong publication type: conference abstract |
| Buechler 2018 | Wrong P: not gone through surgery |
| Bueno-de-Mesquita 2007 | Wrong P: HER2 status missing |
| Chang 2017 | Systematic review, studies with high clinical risk included |
| Chen 2018 | Wrong P: HER2 status missing |
| Chen 2019 | Wrong P: HER2 status missing |
| Cheng 2018 | Wrong O: correlation |
| Cheung 2014 | Wrong O: treatment decision making |
| Chinn-Lenn 2018 | Wrong P: includes both clinical low and high risk breast cancer |
| Curtit 2019 | Wrong P: not clinical intermediate risk of recurrence |
| Cusumano 2014 | Wrong P: includes both clinical low and high risk breast cancer |
| Davidson 2013 | Wrong P: includes too many clinical low risk breast cancer |
| De Boer 2013 | Wrong P: includes too many clinical low risk breast cancer |
| Dieci 2018 | Wrong O: treatment decision making |
| Dinan 2015 | Wrong P: HER2 status unavailable, includes clinical high risk breast cancer |
| Ding 2019 | O missing for I versus C |
| Dreyfus 2015 | Wrong language: French |
| Drukker 2013 | Wrong P: includes HER2-positive and ER-negative |
| Drukker 2014 | Wrong P: includes HER2-positive and ER-negative |
| Drukker 2014 | No C |
| Dzimitrowicz 2017 | Wrong P: includes too many clinical low risk breast cancer, wrong comparison group – controls also receive RS-test. |
| Eichler 2019 | Wrong P: not clinical intermediate breast cancer |
| Eiermann 2013 | Wrong P: includes too many clinical low risk breast cancer |
| Ellis 2016 | Wrong O: treatment decision making |
| Endo 2017 | Wrong publication type: conference abstract |
| EUnetHTA 2018 | Wrong P: includes HER2-positive |
| Evans 2016 | Wrong P: ER- and/or HER2-status missing. Wrong O |
| Exner 2014 | Wrong O: treatment decision making |
| Fallowfield 2018 | Wrong O: treatment decision making |
| Filipits 2019 | I missing: all patients had endocrine treatment only |
| Fried 2014 | Wrong P: HER2 status not available |
| Friese 2017 | Wrong O: treatment decision making |
| Gao 2020 | Wrong O: breast cancer specific survival |
| Geffen 2011 | Wrong P: includes too many clinical low risk breast cancer, HER2 status poorly described |
| Gligorov 2015 | Wrong O: treatment decision making |
| Gluz 2016 | No C |
| Green 2018 | Wrong C |
| Guth 2011 | Wrong I/C: compare hospitals |
| Harris 2016 | Wrong P: population poorly defined |
| Hassett 2012 | Wrong P: includes HER2-positive and N2 |
| Hecquet 2017 | Wrong O: treatment decision making |
| Henry 2009 | Wrong O: treatment decision making |
| Hinde 2019 | Wrong publication type: conference abstract |
| Hochheiser 2019 | Cost-effectiveness study |
| Holt 2013 | Wrong P: includes too many clinical low risk breast cancer, include HER2-positive |
| Hyams 2017 | Wrong publication type: review, not systematic |
| Ibarrondo 2018 | Wrong design: cost-utility analysis |
| Jaafar 2014 | Wrong O: treatment decision making |
| Jankowitz 2011 | No outcome for the patient group at issue |
| Jasem 2016 | Wrong P: includes too many clinical high risk breast cancer |
| Jasem 2017 | No C |
| Joh 2011 | Wrong P: HER2-positive included |
| Katz 2018 | Wrong O: worry after treatment |
| King 2016 | No C |
| Kizy 2017 | Wrong P: includes only lobular carcinoma |
| Kizy 2019 | Wrong P: high-risk RS for comparisons chemotherapy vs no chemotherapy |
| Klang 2010 | Wrong P: HER2 status missing |
| Krijgsman 2012 | Wrong I: test to categorise in subgroups |
| Krop 2017 | Wrong publication type: guidelines |
| Kuchel 2016 | Wrong O: treatment decision making |
| Kuijer 2016 | Wrong O: treatment decision making |
| Kuijer 2016b | Reports two different time periods |
| Kuijer 2017 | Wrong P: includes too many clinical low risk breast cancer |
| Larson 2018 | Wrong O: unclear relationship between test and outcome |
| Lee 2015 | Wrong P: includes too many clinical low risk breast cancer |
| Leung 2016 | Wrong P: includes too many clinical low risk breast cancer |
| Levine 2016 | Wrong P: includes too many both clinical low and high risk breast cancer |
| Li 2017 | Wrong I: to develop a treatment model |
| Liu 2020 | Wrong P: HER2 status missing |
| Lo 2010 | Wrong P: HER2-positive included |
| Loncaster 2017 | Wrong O: treatment decision making |
| Luo 2020 | Wrong O: treatment decision making |
| Lyman 2007 | Economic analysis, unclear description of population |
| Malo 2012 | Wrong O: treatment decision making |
| Mamounas 2018 | Wrong publication type: review, not systematic |
| Marcinkowski 2017 | No C |
| Markopoulos 2012 | Wrong O: treatment decision making |
| Martín 2015 | Wrong O: treatment decision making |
| Martínez del Prado 2018 | Wrong O: treatment decision making |
| McVeigh 2014 | Wrong P: includes too many clinical low risk breast cancer |
| Mokbel 2017 | Wrong O: treatment decision making |
| Muller 2013 | Wrong P: includes both clinical low and high risk breast cancer |
| Narain 2017 | Wrong design, the report investigates the gene expressions tests as a prediction tool for adjunct chemotherapy and recurrence |
| Nguyen 2014 | Wrong O: treatment decision making |
| Nitz 2015 | Wrong publication type: conference abstract |
| Nitz 2017 | Wrong P: includes N2-N3. Wrong comparison |
| Nitz 2019 | C missing (all patients had chemotherapy) |
| Ohnstad 2017 | No C |
| Oratz 2007 | Wrong O: treatment decision making |
| Ozmen 2016 | Wrong P: includes too many both clinical low and high risk breast cancer |
| Panousis 2016 | Wrong O: treatment decision making |
| Park 2018 | Wrong O: treatment decision making |
| Parsons 2016 | Wrong O: treatment decision making |
| Partin 2011 | Wrong O: treatment decision making |
| Peethambaram 2017 | Wrong P: includes too many clinical high risk breast cancer |
| Penault-Llorca 2020 | Wrong O: treatment decision making, anxiety, distress |
| Pestalozzi 2017 | Wrong P: includes too many both clinical low and high risk breast cancer |
| Petkov 2016 | No C |
| Plitcha 2018 | Wrong C: 7th and 8th edition of AJCC staging manual |
| Pohl 2016 | Wrong P: intermediate clinical risk not defined |
| Poorvu 2020 | No comparison with/without test. Wrong P for comparison with/without chemotherapy (not restricted to low/intermediate genetic risk) |
| Potosky 2015 | Wrong O: treatment decision making |
| Prat 2019 | Wrong P: includes metastatic disease |
| Pu 2020 | Wrong P: HER2 positive |
| Ray 2016 | Wrong P: includes too many clinical low risk breast cancer |
| Rayhanabad 2008 | Wrong O: treatment decision making |
| Retel 2013 | No C |
| Reyes 2019 | Wrong O: treatment decision making |
| Roberts 2017 | Wrong P: wrong comparison |
| Sanchez-Forgach 2017 | Wrong language: Spanish |
| Sanft 2015 | Wrong O: extended endocrine therapy |
| Schneider 2012 | Wrong O: treatment decision making |
| Schreuder 2017 | Wrong P: includes too many both clinical low and high risk breast cancer |
| Schreuder 2018 | Wrong O: treatment decision making |
| Scope 2017 | Systematic review, studies with high clinical risk included |
| Sestak 2018 | No C |
| Shimizu 2015 | No C |
| Siegelmann 2013 | Wrong O: treatment decision making |
| Smyth 2015 | Wrong P: HER2 status missing |
| Sparano 2015 | No C |
| Stemmer 2013 | Wrong P: HER2 status missing in control group |
| Sulayman 2012 | No C |
| Tang 2011 | Wrong P: HER2 status missing |
| Torres 2018 | Wrong O: treatment decision making |
| Torrisi 2013 | Wrong P: includes too many clinical low and high risk breast cancer |
| Tsai 2018 | Wrong C: Mammaprint versus Oncotype DX |
| Turashvili 2018 | No C |
| Turner 2019 | Wrong focus: validation of algorithm against Oncotype DX |
| Tzeng 2010 | No C |
| Waintraub 2017 | Wrong O: treatment decision making |
| van 't Veer 2002 | Wrong publication type: letter |
| Wang 2019 | Wrong design: cost-effectiveness analysis |
| Voelker 2018 | Wrong O: treatment decision making |
| Wu 2020 | Wrong I/C: results according to RS score |
| Wuerstlein 2016 | Wrong O: treatment decision making |
| Xiang 2019 | Wrong P: multigene testing |
| Xiao 2017 | Wrong C |
| Yamauchi 2014 | Wrong P: includes too many clinical low risk breast cancer |
| Zeng 2017 | Wrong O: treatment decision making |
| Zhang 2015 | Wrong O: treatment decision making |

**Articles excluded after full-text reading, full reference**

1. Aalders KC, Kuijer A, Straver ME, Slaets L, Litiere S, Viale G, et al. Characterisation of multifocal breast cancer using the 70-gene signature in clinical low-risk patients enrolled in the EORTC 10041/BIG 03-04 MINDACT trial. European Journal of Cancer. 2017;79:98-105.
2. Adamo B, Bellet M, Pare L, Pascual T, Vidal M, Perez Fidalgo JA, et al. Oral metronomic vinorelbine combined with endocrine therapy in hormone receptor-positive HER2-negative breast cancer: SOLTI-1501 VENTANA window of opportunity trial. Breast Cancer Research. 2019;21(1):108.
3. Ademuyiwa FO, Miller A, O'Connor T, Edge SB, Thorat MA, Sledge GW, et al. The effects of oncotype DX recurrence scores on chemotherapy utilization in a multi-institutional breast cancer cohort. Breast Cancer Research & Treatment. 2011;126(3):797-802.
4. Albanell J, Gonzalez A, Ruiz-Borrego M, Alba E, Garcia-Saenz JA, Corominas JM, et al. Prospective transGEICAM study of the impact of the 21-gene Recurrence Score assay and traditional clinicopathological factors on adjuvant clinical decision making in women with estrogen receptor-positive (ER+) node-negative breast cancer. Annals of Oncology. 2012;23(3):625-31.
5. Albanell J, Svedman C, Gligorov J, Holt SD, Bertelli G, Blohmer JU, et al. Pooled analysis of prospective European studies assessing the impact of using the 21-gene Recurrence Score assay on clinical decision making in women with oestrogen receptor-positive, human epidermal growth factor receptor 2-negative early-stage breast cancer. European Journal of Cancer. 2016;66:104-13.
6. Altman AM, Marmor S, Tuttle TM, Hui JYC. 21-Gene Recurrence Score Testing in HER2-positive Patients. Clin Breast Cancer. 2018.
7. Amro A, Chen Y, Barry R, Susick L, Bensenhaver J, Proctor E, et al. Distribution and Short-term Prognostic Value of the 21-gene recurrence score in African American compared to White American breast cancer patients. Breast Journal. 2019;25(4):667-71.
8. Arima N, Nishimura R, Osako T, Okumura Y, Nakano M, Fujisue M, et al. Ki-67 index value and progesterone receptor status can predict prognosis and suitable treatment in node-negative breast cancer patients with estrogen receptor-positive and HER2-negative tumors. Oncol Lett. 2019;17(1):616-22.
9. Asad J, Jacobson AF, Estabrook A, Smith SR, Boolbol SK, Feldman SM, et al. Does oncotype DX recurrence score affect the management of patients with early-stage breast cancer? American Journal of Surgery. 2008;196(4):527-9.
10. Baitchu Y, Apffelstaedt J. Application of Gene Profiling in Selection of Adjuvant Therapy in Breast Cancer in a Developing Country. South African Journal of Surgery. 2017;55(3):71-2.
11. Bargallo JE, Lara F, Shaw-Dulin R, Perez-Sanchez V, Villarreal-Garza C, Maldonado-Martinez H, et al. A study of the impact of the 21-gene breast cancer assay on the use of adjuvant chemotherapy in women with breast cancer in a Mexican public hospital. Journal of Surgical Oncology. 2015;111(2):203-7.
12. Bartlett JMS, Sgroi DC, Treuner K, Zhang Y, Ahmed I, Piper T, et al. Breast Cancer Index and prediction of benefit from extended endocrine therapy in breast cancer patients treated in the Adjuvant Tamoxifen-To Offer More? (aTTom) trial. Annals of Oncology. 2019;30(11):1776-83.
13. Bear HD, Wan W, Robidoux A, Rubin P, Limentani S, White RL, Jr., et al. Using the 21-gene assay from core needle biopsies to choose neoadjuvant therapy for breast cancer: A multicenter trial. Journal of Surgical Oncology. 2017;115(8):917-23.
14. Bhargava R, Clark BZ, Carter GJ, Brufsky AM, Dabbs DJ. The healthcare value of the Magee Decision Algorithm TM: use of Magee Equations TM and mitosis score to safely forgo molecular testing in breast cancer. Modern Pathology. 2020;17:17.
15. Bhutiani N, Vuong B, Egger ME, Eldredge-Hindy H, McMasters KM, Ajkay N. Evaluating patterns of utilization of gene signature panels and impact on treatment patterns in patients with ductal carcinoma in situ of the breast. Surgery. 2019;166(4):509-14.
16. Biroschak JR, Schwartz GF, Palazzo JP, Toll AD, Brill KL, Jaslow RJ, et al. Impact of Oncotype DX on treatment decisions in ER-positive, node-negative breast cancer with histologic correlation. Breast Journal. 2013;19(3):269-75.
17. Blok EJ, Bastiaannet E, van den Hout WB, Liefers GJ, Smit V, Kroep JR, et al. Systematic review of the clinical and economic value of gene expression profiles for invasive early breast cancer available in Europe. Cancer Treatment Reviews. 2018;62:74-90.
18. Bonneterre J, Prat A, Galvan P, Morel P, Giard S. Value of a gene signature assay in patients with early breast cancer and intermediate risk: a single institution retrospective study. Current Medical Research & Opinion. 2016;32(5):835-9.
19. Bradbury AR, Egleston BL, Patrick-Miller LJ, Rustgi N, Brandt A, Brower J, et al. Longitudinal outcomes with cancer multigene panel testing in previously tested BRCA1/2 negative patients. Clinical Genetics. 2020;97(4):601-9.
20. Breaux A, Turner B, Wu X, Rai SN, Riley EC, Mandadi M, et al. Impact of 21-Gene Expression Assay on Staging Estrogen Receptor-Positive HER2-Negative Breast Cancer. Clinical Breast Cancer. 2018;29:29.
21. Brownlie D, Chatterjee S, Saad Z, Wilson G, Bashir M. Impact of Oncotype DX on the decision for adjuvant chemotherapy: retrospective analysis of the Salford Royal Foundation Trust cohort. European journal of surgical oncology. 2015;41(6):S74.
22. Buechler SA, Gokmen-Polar Y, Badve SS. EarlyR signature predicts response to neoadjuvant chemotherapy in breast cancer. Breast. 2018;43:74-80.
23. Bueno-de-Mesquita JM, van Harten WH, Retel VP, van 't Veer LJ, van Dam FS, Karsenberg K, et al. Use of 70-gene signature to predict prognosis of patients with node-negative breast cancer: a prospective community-based feasibility study (RASTER). Erratum appears in Lancet Oncol. 2008 Jan;9(1):10. Lancet Oncology. 2007;8(12):1079-87.
24. Chang MC, Souter LH, Kamel-Reid S, Rutherford M, Bedard P, Trudeau M, et al. Clinical utility of multigene profiling assays in early-stage breast cancer. Current Oncology. 2017;24(5):e403-e22.
25. Chen WR, Deng JP, Wang J, Sun JY, He ZY, Wu SG. Impact of 21-Gene Recurrence Score on Chemotherapy Decision in Invasive Ductal Carcinoma of Breast with Nodal Micrometastases. Cancer Research & Treatment. 2019;51(4):1437-48.
26. Chen XH, Zhang WW, Wang J, Sun JY, Li FY, He ZY, et al. 21-gene recurrence score and adjuvant chemotherapy decisions in patients with invasive lobular breast cancer. Biomark Med. 2018.
27. Cheng V, Markarian A, de Lemos ML, Schaff K. Evaluation of the role of pharmacy technicians in reviewing the eligibility for Oncotype DX genomic test and the impact of the test on treatment plans in breast cancer patients. J Oncol Pharm Pract. 2018:1078155218803703.
28. Cheung PS, Tong AC, Leung RC, Kwan WH, Yau TC. Initial experience with the Oncotype DX assay in decision-making for adjuvant therapy of early oestrogen receptor-positive breast cancer in Hong Kong. Hong Kong Medical Journal. 2014;20(5):401-6.
29. Chin-Lenn L, De Boer RH, Segelov E, Marx GM, Hughes TM, McCarthy NJ, et al. The impact and indications for Oncotype DX on adjuvant treatment recommendations when third-party funding is unavailable. Asia Pac J Clin Oncol. 2018;14(6):410-6.
30. Curtit E, Vannetzel JM, Darmon JC, Roche S, Bourgeois H, Dewas S, et al. Results of PONDx, a prospective multicenter study of the Oncotype DX((R)) breast cancer assay: Real-life utilization and decision impact in French clinical practice. Breast. 2019;44:39-45.
31. Cusumano PG, Generali D, Ciruelos E, Manso L, Ghanem I, Lifrange E, et al. European inter-institutional impact study of MammaPrint. Breast. 2014;23(4):423-8.
32. Davidson JA, Cromwell I, Ellard SL, Lohrisch C, Gelmon KA, Shenkier T, et al. A prospective clinical utility and pharmacoeconomic study of the impact of the 21-gene Recurrence Score assay in oestrogen receptor positive node negative breast cancer. European Journal of Cancer. 2013;49(11):2469-75.
33. de Boer RH, Baker C, Speakman D, Chao CY, Yoshizawa C, Mann GB. The impact of a genomic assay (Oncotype DX) on adjuvant treatment recommendations in early breast cancer. Medical Journal of Australia. 2013;199(3):205-8.
34. Dieci MV, Guarneri V, Giarratano T, Mion M, Tortora G, De Rossi C, et al. First Prospective Multicenter Italian Study on the Impact of the 21-Gene Recurrence Score in Adjuvant Clinical Decisions for Patients with ER Positive/HER2 Negative Breast Cancer. Oncologist. 2018;23(3):297-305.
35. Dinan MA, Mi X, Reed SD, Lyman GH, Curtis LH. Association Between Use of the 21-Gene Recurrence Score Assay and Receipt of Chemotherapy Among Medicare Beneficiaries With Early-Stage Breast Cancer, 2005-2009. JAMA Oncology. 2015;1(8):1098-109.
36. Ding S, Wu J, Lin C, Andriani L, Goh C, Chen W, et al. Evaluation of the Incorporation of Recurrence Score into the American Joint Committee on Cancer Eighth Edition Staging System in Patients with T1-2N0M0, Estrogen Receptor-Positive, Human Epidermal Growth Receptor 2-Negative Invasive Breast Cancer: A Population-Based Analysis. Oncologist. 2019;24(11):e1014-e23.
37. Dreyfus C, Ballester M, Gligorov J, Agranat P, Antoine M, Tengher I, et al. Impact of the 21-gene assay in decision-making during multidisciplinary breast meeting: A French experience. Gynecologie Obstetrique et Fertilite. 2015;43(12):780-5.
38. Drukker CA, Bueno-de-Mesquita JM, Retel VP, van Harten WH, van Tinteren H, Wesseling J, et al. A prospective evaluation of a breast cancer prognosis signature in the observational RASTER study. International Journal of Cancer. 2013;133(4):929-36.
39. Drukker CA, Nijenhuis MV, Bueno-de-Mesquita JM, Retel VP, van Harten WH, van Tinteren H, et al. Optimized outcome prediction in breast cancer by combining the 70-gene signature with clinical risk prediction algorithms. Breast Cancer Research & Treatment. 2014;145(3):697-705.
40. Drukker CA, van Tinteren H, Schmidt MK, Rutgers EJ, Bernards R, van de Vijver MJ, et al. Long-term impact of the 70-gene signature on breast cancer outcome. Breast Cancer Research & Treatment. 2014;143(3):587-92.
41. Dzimitrowicz H, Mougalian S, Storms S, Hurd S, Chagpar AB, Killelea BK, et al. Impacts of Early Guideline-Directed 21-Gene Recurrence Score Testing on Adjuvant Therapy Decision Making. J Oncol Pract. 2017;13(12):e1012-e20.
42. Eichler C, Fromme J, Thangarajah F, Puppe J, Paepke S, Warm M, et al. Gene-expression Profiling - A Decision Impact Analysis: Decision Dependency on Oncotype DX as a Function of Oncological Work Experience in 117 Cases. Anticancer Research. 2019;39(1):297-303.
43. Eiermann W, Rezai M, Kummel S, Kuhn T, Warm M, Friedrichs K, et al. The 21-gene recurrence score assay impacts adjuvant therapy recommendations for ER-positive, node-negative and node-positive early breast cancer resulting in a risk-adapted change in chemotherapy use. Annals of Oncology. 2013;24(3):618-24.
44. Ellis PG, Brufsky AM, Beriwal S, Lokay KG, Benson HO, McCutcheon SB, et al. Pathways Clinical Decision Support for Appropriate Use of Key Biomarkers. J Oncol Pract. 2016;12(6):e681-7.
45. Endo Y, Dong Y, Kondo N, Hato Y, Hisada T, Nishimoto M, et al. Exome sequencing of human breast cancer tissues resistant to taxanes. Cancer research Conference: 39th annual CTRC-AACR san antonio breast cancer symposium United states. 2017;77(4).
46. EUnetHTA Joint Action 3 WP4. Added value of using the gene expression signature test MammaPrint for adjuvant chemotherapy decision-making in early breast cancer. European Network for Health Technology Assessment. 2018.
47. Evans CN, Brewer NT, Vadaparampil ST, Boisvert M, Ottaviano Y, Lee MC, et al. Impact of genomic testing and patient-reported outcomes on receipt of adjuvant chemotherapy. Breast Cancer Research & Treatment. 2016;156(3):549-55.
48. Exner R, Bago-Horvath Z, Bartsch R, Mittlboeck M, Retel VP, Fitzal F, et al. The multigene signature MammaPrint impacts on multidisciplinary team decisions in ER+, HER2- early breast cancer. British Journal of Cancer. 2014;111(5):837-42.
49. Fallowfield L, Matthews L, May S, Jenkins V, Bloomfield D. Enhancing decision-making about adjuvant chemotherapy in early breast cancer following EndoPredict testing. Psychooncology. 2018;27(4):1264-9.
50. Filipits M, Dubsky P, Rudas M, Greil R, Balic M, Bago-Horvath Z, et al. Prediction of distant recurrence using EndoPredict among women with ER+, HER2-node-positive and node-negative breast cancer treated with endocrine therapy only. Clinical Cancer Research. 2019;25(13):3865-72.
51. Fried G, Moskovitz M. Treatment decisions in estrogen receptor-positive early breast cancer patients with intermediate oncotype DX recurrence score results. Springerplus. 2014;3:71.
52. Friese CR, Li Y, Bondarenko I, Hofer TP, Ward KC, Hamilton AS, et al. Chemotherapy decisions and patient experience with the recurrence score assay for early-stage breast cancer. Cancer. 2017;123(1):43-51.
53. Gao W, Lin L, Fei X, Chen X, Shen K. Decision-making of Adjuvant Chemotherapy for Breast Cancer Patients with Discordant Risk Classifications between Clinical-Pathological Factors and 21-gene Recurrence Score. Journal of Cancer. 2020;11(9):2509-17.
54. Geffen DB, Abu-Ghanem S, Sion-Vardy N, Braunstein R, Tokar M, Ariad S, et al. The impact of the 21-gene recurrence score assay on decision making about adjuvant chemotherapy in early-stage estrogen-receptor-positive breast cancer in an oncology practice with a unified treatment policy. Annals of Oncology. 2011;22(11):2381-6.
55. Gligorov J, Pivot XB, Jacot W, Naman HL, Spaeth D, Misset JL, et al. Prospective Clinical Utility Study of the Use of the 21-Gene Assay in Adjuvant Clinical Decision Making in Women With Estrogen Receptor-Positive Early Invasive Breast Cancer: Results From the SWITCH Study. Oncologist. 2015;20(8):873-9.
56. Gluz O, Nitz UA, Christgen M, Kates RE, Shak S, Clemens M, et al. West German Study Group Phase III PlanB Trial: First Prospective Outcome Data for the 21-Gene Recurrence Score Assay and Concordance of Prognostic Markers by Central and Local Pathology Assessment. Journal of Clinical Oncology. 2016;34(20):2341-9.
57. Green N, Al-Allak A, Fowler C. Benefits of introduction of Oncotype DX® testing. Annals of the Royal College of Surgeons of England. 2019;101(1):55-9.
58. Guth AA, Fineberg S, Fei K, Franco R, Bickell N. Utilization of Oncotype DX to predict chemotherapy use in an inner-city population. Journal of Clinical Oncology. 2011;29(15).
59. Harris LN, Ismaila N, McShane LM, Andre F, Collyar DE, Gonzalez-Angulo AM, et al. Use of Biomarkers to Guide Decisions on Adjuvant Systemic Therapy for Women With Early-Stage Invasive Breast Cancer: American Society of Clinical Oncology Clinical Practice Guideline. Journal of Clinical Oncology. 2016;34(10):1134-50.
60. Hassett MJ, Silver SM, Hughes ME, Blayney DW, Edge SB, Herman JG, et al. Adoption of gene expression profile testing and association with use of chemotherapy among women with breast cancer. Journal of Clinical Oncology. 2012;30(18):2218-26.
61. Henry LR, Stojadinovic A, Swain SM, Prindiville S, Cordes R, Soballe PW. The influence of a gene expression profile on breast cancer decisions. Journal of Surgical Oncology. 2009;99(6):319-23.
62. Hequet D, Callens C, Gentien D, Albaud B, Mouret-Reynier MA, Dubot C, et al. Prospective, multicenter French study evaluating the clinical impact of the Breast Cancer Intrinsic Subtype-Prosigna Test in the management of early-stage breast cancers. PLoS ONE [Electronic Resource]. 2017;12(10):e0185753.
63. Hinde S, Theriou C, May S, Matthews L, Arbon A, Fallowfield L, et al. The cost-effectiveness of EndoPredict to inform adjuvant chemotherapy decisions in early breast cancer. Health Policy and Technology. 2019.
64. Hochheiser L, Hornberger J, Turner M, Lyman GH. Multi-gene assays: effect on chemotherapy use, toxicity and cost in estrogen receptor-positive early stage breast cancer. J Comp Eff Res. 2019.
65. Holt S, Bertelli G, Humphreys I, Valentine W, Durrani S, Pudney D, et al. A decision impact, decision conflict and economic assessment of routine Oncotype DX testing of 146 women with node-negative or pNImi, ER-positive breast cancer in the U.K. British Journal of Cancer. 2013;108(11):2250-8.
66. Hyams DM, Schuur E, Angel Aristizabal J, Bargallo Rocha JE, Cabello C, Elizalde R, et al. Selecting postoperative adjuvant systemic therapy for early stage breast cancer: A critical assessment of commercially available gene expression assays. Journal of Surgical Oncology. 2017;115(6):647-62.
67. Ibarrondo O, Alvarez-Lopez I, Freundlich F, Arrospide A, Galve-Calvo E, Gutierrez-Toribio M, et al. Probabilistic cost-utility analysis and expected value of perfect information for the Oncotype multigenic test: a discrete event simulation model. Gac Sanit. 2018.
68. Jaafar H, Bashir MA, Taher A, Qawasmeh K, Jaloudi M. Impact of Oncotype DX testing on adjuvant treatment decisions in patients with early breast cancer: a single-center study in the United Arab Emirates. Asia-Pacific Journal of Clinical Oncology. 2014;10(4):354-60.
69. Jankowitz RC, Cooper K, Erlander MG, Ma XJ, Kesty NC, Li H, et al. Prognostic utility of the breast cancer index and comparison to Adjuvant! Online in a clinical case series of early breast cancer. Breast Cancer Research. 2011;13(5):R98.
70. Jasem J, Amini A, Rabinovitch R, Borges VF, Elias A, Fisher CM, et al. 21-Gene Recurrence Score Assay As a Predictor of Adjuvant Chemotherapy Administration for Early-Stage Breast Cancer: An Analysis of Use, Therapeutic Implications, and Disparity Profile. Journal of Clinical Oncology. 2016;34(17):1995-2002.
71. Jasem J, Fisher CM, Amini A, Shagisultanova E, Rabinovitch R, Borges VF, et al. The 21-Gene Recurrence Score Assay for Node-Positive, Early-Stage Breast Cancer and Impact of RxPONDER Trial on Chemotherapy Decision-Making: Have Clinicians Already Decided? Journal of the National Comprehensive Cancer Network. 2017;15(4):494-503.
72. Joh JE, Esposito NN, Kiluk JV, Laronga C, Lee MC, Loftus L, et al. The effect of Oncotype DX recurrence score on treatment recommendations for patients with estrogen receptor-positive early stage breast cancer and correlation with estimation of recurrence risk by breast cancer specialists. Oncologist. 2011;16(11):1520-6.
73. Katz SJ, Ward KC, Hamilton AS, Abrahamse P, Hawley ST, Kurian AW. Association of Germline Genetic Test Type and Results With Patient Cancer Worry After Diagnosis of Breast Cancer. JCO Precis Oncol. 2018;2018.
74. King TA, Lyman JP, Gonen M, Voci A, De Brot M, Boafo C, et al. Prognostic Impact of 21-Gene Recurrence Score in Patients With Stage IV Breast Cancer: TBCRC 013. Journal of Clinical Oncology. 2016;34(20):2359-65.
75. Kizy S, Altman AM, Marmor S, Denbo JW, Jensen EH, Tuttle TM, et al. 21-gene recurrence score testing in the older population with estrogen receptor-positive breast cancer. Journal of Geriatric Oncology. 2019;10(2):322-9.
76. Kizy S, Huang JL, Marmor S, Tuttle TM, Hui JYC. Impact of the 21-gene recurrence score on outcome in patients with invasive lobular carcinoma of the breast. Breast Cancer Research & Treatment. 2017;165(3):757-63.
77. Klang SH, Hammerman A, Liebermann N, Efrat N, Doberne J, Hornberger J. Economic implications of 21-gene breast cancer risk assay from the perspective of an Israeli-managed health-care organization. Value in Health. 2010;13(4):381-7.
78. Krijgsman O, Roepman P, Zwart W, Carroll JS, Tian S, de Snoo FA, et al. A diagnostic gene profile for molecular subtyping of breast cancer associated with treatment response. Breast Cancer Research & Treatment. 2012;133(1):37-47.
79. Krop I, Ismaila N, Andre F, Bast RC, Barlow W, Collyar DE, et al. Use of Biomarkers to Guide Decisions on Adjuvant Systemic Therapy for Women With Early-Stage Invasive Breast Cancer: American Society of Clinical Oncology Clinical Practice Guideline Focused Update. Journal of Clinical Oncology. 2017;35(24):2838-47.
80. Kuchel A, Robinson T, Comins C, Shere M, Varughese M, Sparrow G, et al. The impact of the 21-gene assay on adjuvant treatment decisions in oestrogen receptor-positive early breast cancer: a prospective study. British Journal of Cancer. 2016;114(7):731-6.
81. Kuijer A, Drukker CA, Elias SG, Smorenburg CH, Th. Rutgers EJ, Siesling S, et al. Changes over time in the impact of gene-expression profiles on the administration of adjuvant chemotherapy in estrogen receptor positive early stage breast cancer patients: A nationwide study. International Journal of Cancer. 2016b;139(4):769-75.
82. Kuijer A, Straver M, den Dekker B, van Bommel ACM, Elias SG, Smorenburg CH, et al. Impact of 70-Gene Signature Use on Adjuvant Chemotherapy Decisions in Patients With Estrogen Receptor-Positive Early Breast Cancer: Results of a Prospective Cohort Study. Journal of Clinical Oncology. 2017;35(24):2814-9.
83. Kuijer A, van Bommel AC, Drukker CA, van der Heiden-van der Loo M, Smorenburg CH, Westenend PJ, et al. Using a gene expression signature when controversy exists regarding the indication for adjuvant systemic treatment reduces the proportion of patients receiving adjuvant chemotherapy: a nationwide study. Genetics in Medicine. 2016a;18(7):720-6.
84. Larson KE, Valente SA, Shah C, Tendulkar RD, Cherian S, Abraham J, et al. Oncotype testing in patients undergoing intraoperative radiation for breast cancer. Mol Clin Oncol. 2018;9(5):535-8.
85. Lee MH, Han W, Lee JE, Kim KS, Park H, Kim J, et al. The clinical impact of 21-gene recurrence score on treatment decisions for patients with hormone receptor-positive early breast cancer in Korea. Cancer Research & Treatment. 2015;47(2):208-14.
86. Leung RC, Yau TC, Chan MC, Chan SW, Chan TW, Tsang YY, et al. The Impact of the Oncotype DX Breast Cancer Assay on Treatment Decisions for Women With Estrogen Receptor-Positive, Node-Negative Breast Carcinoma in Hong Kong. Clinical Breast Cancer. 2016;16(5):372-8.
87. Levine MN, Julian JA, Bedard PL, Eisen A, Trudeau ME, Higgins B, et al. Prospective Evaluation of the 21-Gene Recurrence Score Assay for Breast Cancer Decision-Making in Ontario. Journal of Clinical Oncology. 2016;34(10):1065-71.
88. Li Y, Kurian AW, Bondarenko I, Taylor JMG, Jagsi R, Ward KC, et al. The influence of 21-gene recurrence score assay on chemotherapy use in a population-based sample of breast cancer patients. Breast Cancer Research & Treatment. 2017;161(3):587-95.
89. Liu KH, Zhang L, Chen JX, Lian CL, Wang J, He ZY, et al. Should women with early breast cancer under 40 years of age have a routine 21-gene recurrence score testing: A SEER database study. Breast. 2020;49:233-41.
90. Lo SS, Mumby PB, Norton J, Rychlik K, Smerage J, Kash J, et al. Prospective multicenter study of the impact of the 21-gene recurrence score assay on medical oncologist and patient adjuvant breast cancer treatment selection. Journal of Clinical Oncology. 2010;28(10):1671-6.
91. Loncaster J, Armstrong A, Howell S, Wilson G, Welch R, Chittalia A, et al. Impact of Oncotype DX breast Recurrence Score testing on adjuvant chemotherapy use in early breast cancer: Real world experience in Greater Manchester, UK. Erratum appears in Eur J Surg Oncol. 2017 Nov 23;:; PMID: 29174199. European Journal of Surgical Oncology. 2017;43(5):931-7.
92. Luo M, Li F, Su K, Yuan H, Zeng J. Comparison of 21-gene assay and St.Gallen International Expert Consensus in the treatment decision for patients with early invasive breast cancers. Cancer Biology & Therapy. 2020;21(2):108-12.
93. Lyman GH, Cosler LE, Kuderer NM, Hornberger J. Impact of a 21-gene RT-PCR assay on treatment decisions in early-stage breast cancer: an economic analysis based on prognostic and predictive validation studies. Cancer. 2007;109(6):1011-8.
94. Malo TL, Lipkus I, Wilson T, Han HS, Acs G, Vadaparampil ST. Treatment Choices Based on OncotypeDx in the Breast Oncology Care Setting. Journal of Cancer Epidemiology Print. 2012;2012:941495.
95. Mamounas EP, Russell CA, Lau A, Turner MP, Albain KS. Clinical relevance of the 21-gene Recurrence Score((R)) assay in treatment decisions for patients with node-positive breast cancer in the genomic era. NPJ Breast Cancer. 2018;4:27.
96. Marcinkowski EF, Ottesen R, Niland J, Vito C. Acceptance of adjuvant chemotherapy recommendations in early-stage hormone-positive breast cancer. Journal of Surgical Research. 2017;214:79-85.
97. Markopoulos C, Xepapadakis G, Venizelos V, Tsiftsoglou A, Misitzis J, Panoussis D, et al. Clinical experience of using Oncotype DX as an additional treatment decision tool in early breast cancer - a retrospective analysis from 5 Greek institutions. European Journal of Surgical Oncology. 2012;38(5):413-9.
98. Martin M, Gonzalez-Rivera M, Morales S, de la Haba-Rodriguez J, Gonzalez-Cortijo L, Manso L, et al. Prospective study of the impact of the Prosigna assay on adjuvant clinical decision-making in unselected patients with estrogen receptor positive, human epidermal growth factor receptor negative, node negative early-stage breast cancer. Current Medical Research & Opinion. 2015;31(6):1129-37.
99. Martinez Del Prado P, Alvarez-Lopez I, Dominguez-Fernandez S, Plazaola A, Ibarrondo O, Galve-Calvo E, et al. Clinical and economic impact of the 21-gene recurrence score assay in adjuvant therapy decision making in patients with early-stage breast cancer: pooled analysis in 4 Basque Country university hospitals. Clinicoecon Outcomes Res. 2018;10:189-99.
100. McVeigh TP, Hughes LM, Miller N, Sheehan M, Keane M, Sweeney KJ, et al. The impact of Oncotype DX testing on breast cancer management and chemotherapy prescribing patterns in a tertiary referral centre. European Journal of Cancer. 2014;50(16):2763-70.
101. Mokbel K, Wazir U, El Hage Chehade H, Manson A, Choy C, Moye V, et al. A Comparison of the Performance of EndoPredict Clinical and NHS PREDICT in 120 Patients Treated for ER-positive Breast Cancer. Anticancer Research. 2017;37(12):6863-9.
102. Muller BM, Keil E, Lehmann A, Winzer KJ, Richter-Ehrenstein C, Prinzler J, et al. The EndoPredict Gene-Expression Assay in Clinical Practice - Performance and Impact on Clinical Decisions. PLoS One. 2013;8(6):e68252.
103. Narain T, Adcock L. Gene Expression Tests for Women with Early Stage Breast Cancer: A Review of Clinical Utility and Cost-Effectiveness. Ottawa (ON): Canadian Agency for Drugs and Technologies in Health; 2017.
104. Nguyen MT, Stessin A, Nagar H, D'Alfonso TM, Chen Z, Cigler T, et al. Impact of oncotype DX recurrence score in the management of breast cancer cases. Clinical Breast Cancer. 2014;14(3):182-90.
105. Nitz U, Gluz O, Christgen M, Kates RE, Clemens M, Malter W, et al. Reducing chemotherapy use in clinically high-risk, genomically low-risk pN0 and pN1 early breast cancer patients: five-year data from the prospective, randomised phase 3 West German Study Group (WSG) PlanB trial. Breast Cancer Research & Treatment. 2017;165(3):573-83.
106. Nitz U, Gluz O, Clemens M, Malter W, Reimer T, Nuding B, et al. West German Study PlanB Trial: Adjuvant Four Cycles of Epirubicin and Cyclophosphamide Plus Docetaxel Versus Six Cycles of Docetaxel and Cyclophosphamide in HER2-Negative Early Breast Cancer. Journal of Clinical Oncology. 2019;37(10):799-808.
107. Nitz U, Gluz O, Kates RE, Hofmann D, Kreipe HH, Christgen M, et al. Prognostic impact of discordance between different risk assessment tools in early breast cancer (recurrence score, central grade, Ki67): early outcome analysis from the prospective phase III WSG-PlanB trial. Cancer research. 2015;75(9).
108. Ohnstad HO, Borgen E, Falk RS, Lien TG, Aaserud M, Sveli MAT, et al. Prognostic value of PAM50 and risk of recurrence score in patients with early-stage breast cancer with long-term follow-up. Breast Cancer Research. 2017;19(1):120.
109. Oratz R, Paul D, Cohn AL, Sedlacek SM. Impact of a commercial reference laboratory test recurrence score on decision making in early-stage breast cancer. Journal of Oncology Practice. 2007;3(4):182-6.
110. Ozmen V, Atasoy A, Gokmen E, Ozdogan M, Guler N, Uras C, et al. Impact of Oncotype DX Recurrence Score on Treatment Decisions: Results of a Prospective Multicenter Study in Turkey. Cureus. 2016;8(3):e522.
111. Panousis D, Ntasiou P, Grosomanidis D, Chatzopoulos K, Paraskevakou G, Kontogianni P, et al. Impact of Oncotype DX on chemotherapy assignment: a retrospective single-center study on female breast cancer patients. Journal of BUOn. 2017;22(5):1199-208.
112. Park SJ, Lee MH, Kong SY, Song MK, Joo J, Kwon Y, et al. Use of adjuvant chemotherapy in hormone receptor-positive breast cancer patients with or without the 21-gene expression assay. Breast Cancer Research & Treatment. 2018;170(1):69-76.
113. Parsons BM, Landercasper J, Smith AL, Go RS, Borgert AJ, Dietrich LL. 21-Gene recurrence score decreases receipt of chemotherapy in ER+ early-stage breast cancer: an analysis of the NCDB 2010-2013. Breast Cancer Research & Treatment. 2016;159(2):315-26.
114. Partin JF, Mamounas EP. Impact of the 21-gene recurrence score assay compared with standard clinicopathologic guidelines in adjuvant therapy selection for node-negative, estrogen receptor-positive breast cancer. Annals of Surgical Oncology. 2011;18(12):3399-406.
115. Peethambaram PP, Hoskin TL, Day CN, Goetz MP, Habermann EB, Boughey JC. Use of 21-gene recurrence score assay to individualize adjuvant chemotherapy recommendations in ER+/HER2- node positive breast cancer-A National Cancer Database study. NPJ Breast Cancer. 2017;3:41.
116. Penault-Llorca F, Kwiatkowski F, Arnaud A, Levy C, Leheurteur M, Uwer L, et al. Decision of adjuvant chemotherapy in intermediate risk luminal breast cancer patients: A prospective multicenter trial assessing the clinical and psychological impact of EndoPredict R (EpClin) use (UCBG 2-14). Breast. 2020;49:132-40.
117. Pestalozzi BC, Tausch C, Dedes KJ, Rochlitz C, Zimmermann S, von Moos R, et al. Adjuvant treatment recommendations for patients with ER-positive/HER2-negative early breast cancer by Swiss tumor boards using the 21-gene recurrence score (SAKK 26/10). BMC Cancer. 2017;17(1):265.
118. Petkov VI, Miller DP, Howlader N, Gliner N, Howe W, Schussler N, et al. Breast-cancer-specific mortality in patients treated based on the 21-gene assay: a SEER population-based study. NPJ Breast Cancer. 2016;2:16017.
119. Plichta JK, Ren Y, Thomas SM, Greenup RA, Fayanju OM, Rosenberger LH, et al. Implications for Breast Cancer Restaging Based on the 8th Edition AJCC Staging Manual. Ann Surg. 2018.
120. Pohl H, Kotze MJ, Grant KA, van der Merwe L, Pienaar FM, Apffelstaedt JP, et al. Impact of MammaPrint on Clinical Decision-Making in South African Patients with Early-Stage Breast Cancer. Breast Journal. 2016;22(4):442-6.
121. Poorvu PD, Gelber SI, Rosenberg SM, Ruddy KJ, Tamimi RM, Collins LC, et al. Prognostic Impact of the 21-Gene Recurrence Score Assay Among Young Women With Node-Negative and Node-Positive ER-Positive/HER2-Negative Breast Cancer. Journal of Clinical Oncology. 2020;38(7):725-33.
122. Potosky AL, O'Neill SC, Isaacs C, Tsai HT, Chao C, Liu C, et al. Population-based study of the effect of gene expression profiling on adjuvant chemotherapy use in breast cancer patients under the age of 65 years. Cancer. 2015;121(22):4062-70.
123. Prat A, Brase JC, Cheng Y, Nuciforo P, Pare L, Pascual T, et al. Everolimus plus Exemestane for Hormone Receptor-Positive Advanced Breast Cancer: A PAM50 Intrinsic Subtype Analysis of BOLERO-2. Oncologist. 2019.
124. Pu M, Messer K, Davies SR, Vickery TL, Pittman E, Parker BA, et al. Research-based PAM50 signature and long-term breast cancer survival. Breast Cancer Research & Treatment. 2020;179(1):197-206.
125. Ray GT, Mandelblatt J, Habel LA, Ramsey S, Kushi LH, Li Y, et al. Breast cancer multigene testing trends and impact on chemotherapy use. American Journal of Managed Care. 2016;22(5):e153-60.
126. Rayhanabad JA, Difronzo LA, Haigh PI, Romero L. Changing paradigms in breast cancer management: introducing molecular genetics into the treatment algorithm. American Surgeon. 2008;74(10):887-90.
127. Retel VP, Groothuis-Oudshoorn CG, Aaronson NK, Brewer NT, Rutgers EJ, van Harten WH. Association between genomic recurrence risk and well-being among breast cancer patients. BMC Cancer. 2013;13:295.
128. Reyes SA, De La Cruz LM, Ru M, Pisapati KV, Port E. Practice Changing Potential of TAILORx: A Retrospective Review of the National Cancer Data Base from 2010 to 2015. Annals of Surgical Oncology. 2019;26(10):3397-408.
129. Roberts MC, Miller DP, Shak S, Petkov VI. Breast cancer-specific survival in patients with lymph node-positive hormone receptor-positive invasive breast cancer and Oncotype DX Recurrence Score results in the SEER database. Breast Cancer Research & Treatment. 2017;163(2):303-10.
130. Sanchez-Forgach ER, Carpinteyro-Espin U, Aleman-Aviles JA, Sanchez-Basurto C. Validation and clinical application of MammaPrint<sup></sup> in patients with breast cancer. Cirugia y Cirujanos (English Edition). 2017;85(4):320-4.
131. Sanft T, Aktas B, Schroeder B, Bossuyt V, DiGiovanna M, Abu-Khalaf M, et al. Prospective assessment of the decision-making impact of the Breast Cancer Index in recommending extended adjuvant endocrine therapy for patients with early-stage ER-positive breast cancer. Breast Cancer Research & Treatment. 2015;154(3):533-41.
132. Schneider JG, Khalil DN. Why does Oncotype DX recurrence score reduce adjuvant chemotherapy use? Breast Cancer Research & Treatment. 2012;134(3):1125-32.
133. Schreuder K, Kuijer A, Bentum S, van Dalen T, Siesling S. Use and Impact of the 21-Gene Recurrence Score in Relation to the Clinical Risk of Developing Metastases in Early Breast Cancer Patients in the Netherlands. Public Health Genomics. 2018;21(1-2):1-8.
134. Schreuder K, Kuijer A, Rutgers EJT, Smorenburg CH, van Dalen T, Siesling S. Impact of gene-expression profiling in patients with early breast cancer when applied outside the guideline directed indication area. European Journal of Cancer. 2017;84:270-7.
135. Scope A, Essat M, Pandor A, Rafia R, Ward SE, Wyld L, et al. Gene Expression Profiling and Expanded Immunohistochemistry Tests to Guide Selection of Chemotherapy Regimens in Breast Cancer Management: A Systematic Review. International Journal of Technology Assessment in Health Care. 2017;33(1):32-45.
136. Sestak I, Buus R, Cuzick J, Dubsky P, Kronenwett R, Denkert C, et al. Comparison of the Performance of 6 Prognostic Signatures for Estrogen Receptor-Positive Breast Cancer: A Secondary Analysis of a Randomized Clinical Trial. JAMA Oncology. 2018;4(4):545-53.
137. Shimizu H, Horimoto Y, Arakawa A, Sonoue H, Kurata M, Kosaka T, et al. Application of a 70-Gene Expression Profile to Japanese Breast Cancer Patients. Breast Care. 2015;10(2):118-22.
138. Siegelmann-Danieli N, Silverman B, Zick A, Beit-Or A, Katzir I, Porath A. The impact of the Oncotype DX Recurrence Score on treatment decisions and clinical outcomes in patients with early breast cancer: the Maccabi Healthcare Services experience with a unified testing policy. Ecancermedicalscience. 2013;7:380.
139. Smyth L, Watson G, Walsh EM, Kelly CM, Keane M, Kennedy MJ, et al. Economic impact of 21-gene recurrence score testing on early-stage breast cancer in Ireland. Breast Cancer Research & Treatment. 2015;153(3):573-82.
140. Sparano JA, Gray RJ, Makower DF, Pritchard KI, Albain KS, Hayes DF, et al. Prospective Validation of a 21-Gene Expression Assay in Breast Cancer. New England Journal of Medicine. 2015;373(21):2005-14.
141. Stemmer SM, Klang SH, Ben-Baruch N, Geffen DB, Steiner M, Soussan-Gutman L, et al. The impact of the 21-gene Recurrence Score assay on clinical decision-making in node-positive (up to 3 positive nodes) estrogen receptor-positive breast cancer patients. Breast Cancer Research & Treatment. 2013;140(1):83-92.
142. Sulayman N, Spellman E, Graves KD, Peshkin BN, Isaacs C, Schwartz MD, et al. Psychosocial and Quality of Life in Women Receiving the 21-Gene Recurrence Score Assay: The Impact of Decision Style in Women with Intermediate RS. Journal of Cancer Epidemiology Print. 2012;2012:728290.
143. Tang G, Shak S, Paik S, Anderson SJ, Costantino JP, Geyer CE, Jr., et al. Comparison of the prognostic and predictive utilities of the 21-gene Recurrence Score assay and Adjuvant! for women with node-negative, ER-positive breast cancer: results from NSABP B-14 and NSABP B-20. Breast Cancer Research & Treatment. 2011;127(1):133-42.
144. Torres S, Trudeau M, Gandhi S, Warner E, Verma S, Pritchard KI, et al. Prospective Evaluation of the Impact of the 21-Gene Recurrence Score Assay on Adjuvant Treatment Decisions for Women with Node-Positive Breast Cancer in Ontario, Canada. Oncologist. 2018;23(7):768-75.
145. Torrisi R, Garcia-Etienne CA, Losurdo A, Morenghi E, Di Tommaso L, Gatzemeier W, et al. Potential impact of the 70-gene signature in the choice of adjuvant systemic treatment for ER positive, HER2 negative tumors: a single institution experience. Breast. 2013;22(4):419-24.
146. Tsai M, Lo S, Audeh W, Qamar R, Budway R, Levine E, et al. Association of 70-Gene Signature Assay Findings With Physicians' Treatment Guidance for Patients With Early Breast Cancer Classified as Intermediate Risk by the 21-Gene Assay. JAMA Oncology. 2018;4(1):e173470.
147. Turashvili G, Brogi E, Morrow M, Dickler M, Norton L, Hudis C, et al. Breast carcinoma with 21-gene recurrence score lower than 18: rate of locoregional recurrence in a large series with clinical follow-up. BMC Cancer. 2018;18(1):42.
148. Turner BM, Gimenez-Sanders MA, Soukiazian A, Breaux AC, Skinner K, Shayne M, et al. Risk stratification of ER-positive breast cancer patients: A multi-institutional validation and outcome study of the Rochester Modified Magee algorithm (RoMMa) and prediction of an Oncotype DX R recurrence score <26. Cancer Medicine. 2019;8(9):4176-88.
149. Tzeng JP, Mayer D, Richman AR, Lipkus I, Han PK, Valle CG, et al. Women's experiences with genomic testing for breast cancer recurrence risk. Cancer. 2010;116(8):1992-2000.
150. Waintraub SE, McNamara D, Graham DMA, Pecora AL, Min J, Wu T, et al. Real-world economic value of a 21-gene assay in early-stage breast cancer. American Journal of Managed Care. 2017;23(12):e416-e20.
151. van 't Veer LJ, Dai H, van de Vijver MJ, He YD, Hart AA, Mao M, et al. Gene expression profiling predicts clinical outcome of breast cancer. Nature. 2002;415(6871):530-6.
152. Wang SY, Chen T, Dang W, Mougalian SS, Evans SB, Gross CP. Incorporating Tumor Characteristics to Maximize 21-Gene Assay Utility: A Cost-Effectiveness Analysis. J Natl Compr Canc Netw. 2019;17(1):39-46.
153. Voelker HU, Frey L, Strehl A, Weigel M. Practical Consequences Resulting from the Analysis of a 21-Multigene Array in the Interdisciplinary Conference of a Breast Cancer Center. International Journal of Breast Cancer. 2018;2018(2047089).
154. Wu J, Ding S, Lin L, Fei X, Lin C, Andriani L, et al. Comparison of the Distribution Pattern of 21-Gene Recurrence Score between Mucinous Breast Cancer and Infiltrating Ductal Carcinoma in Chinese Population: A Retrospective Single-Center Study. Cancer Research & Treatment. 2020;28:28.
155. Wuerstlein R, Sotlar K, Gluz O, Otremba B, von Schumann R, Witzel I, et al. The West German Study Group Breast Cancer Intrinsic Subtype study: a prospective multicenter decision impact study utilizing the Prosigna assay for adjuvant treatment decision-making in estrogen-receptor-positive, HER2-negative early-stage breast cancer. Current Medical Research & Opinion. 2016;32(7):1217-24.
156. Xiang HY, Liu YH, Zhang H, Zhang S, Xin L, Xu L, et al. Clinicopathologic analysis of 722 breast cancer patients who met the inclusion criteria of the TAILORx trial. Chinese Medical Journal. 2019;132(24):2914-9.
157. Xiao G, Meng J, Zhang J, Li G, Du N, Qin S, et al. Clinical Application of Detecting 21-Gene Recurrence Score in Predicating Prognosis and Therapy Response of Patients with Breast Cancer from Two Medical Centers. Cancer Investigation. 2017;35(10):639-46.
158. Yamauchi H, Nakagawa C, Takei H, Chao C, Yoshizawa C, Yagata H, et al. Prospective study of the effect of the 21-gene assay on adjuvant clinical decision-making in Japanese women with estrogen receptor-positive, node-negative, and node-positive breast cancer. Clinical Breast Cancer. 2014;14(3):191-7.
159. Zeng Y, Li Q, Qin T, Li S, Jin L, Wu J, et al. Impact of a 21-Gene Recurrence Score Test on the Choice of Adjuvant Chemotherapy for Hormone Receptor-positive Early-stage Breast Cancer: A Prospective Study. Anticancer Research. 2017;37(8):4539-47.
160. Zhang YN, Zhou YD, Mao F, Sun Q. Impact of the 21-Gene Recurrence Score Assay in adjuvant chemotherapy selection for node-negative, hormone receptor-positive breast cancer in the Chinese population. Neoplasma. 2015;62(4):658-65.
